# Nephropathia Epidemica in Metropolitan Area, Germany

**DOI:** 10.3201/eid1308.061425

**Published:** 2007-08

**Authors:** Sandra S. Essbauer, Jonas Schmidt-Chanasit, Ernst L. Madeja, Wolfgang Wegener, Robert Friedrich, Rasa Petraityte, Kestutis Sasnauskas, Jens Jacob, Judith Koch, Gerhard Dobler, Franz J. Conraths, Martin Pfeffer, Christian Pitra, Rainer G. Ulrich

**Affiliations:** * Bundeswehr Institute of Microbiology, Munich, Germany; † Friedrich-Loeffler-Institut, Wusterhausen, Germany; ‡ Frankfurt University Medical School, Frankfurt am Main, Germany; § City Health Office of Cologne, Cologne, Germany; ¶ Institute of Biotechnology, Vilnius, Lithuania; # Federal Biological Research Centre Institute for Agriculture and Forestry, Münster, Germany; ** Robert Koch-Institut, Berlin, Germany; †† Institute for Zoo Biology and Wildlife Research, Berlin, Germany

**Keywords:** Hantavirus, outbreak, nephropathia epidemica, urban region, bank vole, letter

**To the Editor:** Old World hantaviruses (family *Bunyaviridae*) are rodentborne pathogens that can cause hemorrhagic fever with renal syndrome (HFRS) (*1*). At least 3 different pathogenic hantavirus species have been detected in Europe: *Dobrava-Belgrade virus* (DOBV), *Tula virus,* and *Puumala virus* (PUUV) (*1**–**3*). Most human hantavirus infections in Europe are assigned to PUUV transmitted by bank voles (*Myodes glareolus*, formerly *Clethrionomys glareolus*). Although PUUV is thought to cause a mild form of HFRS, designated as nephropathia epidemica (NE), severe courses have been described with a case-fatality ratio of up to 0.6% (*3*).

Even though human hantavirus infections have sporadically been reported in Germany since 1983 (e.g., *4*–*7*), clinically apparent hantavirus infections (HFRS, NE) did not become notifiable diseases in Germany until 2001. From 2001 through 2004, ≈140 to 240 cases per year have officially been documented in Germany; most were caused by PUUV. Regions endemic for PUUV have been identified in southern Germany, especially the Alb-Danube region (*4*,*6*,*8*). Since 2004, 2 aspects of the situation in Germany have changed. First, the number of clinical cases has increased dramatically to a total of 448 in 2005. Second, hantavirus infections have been observed in regions previously not recognized as endemic for hantaviruses (*9*). An increased number of human cases were also observed in other European countries (*10*).

Here we report the first, to our knowledge, documented PUUV-associated urban NE outbreak, which occurred in a city park in Germany. In 2005, a total of 89 cases were reported in the district of Cologne with 41 cases recorded in the city center (incidence 4.2/100 000). In the past, (2001–2004), 3–22 cases were reported annually for the district of Cologne and 2–6 cases for the city of Cologne. Clinical symptoms, documented by responses to a questionnaire, resembled those typical for NE found in previous studies in Germany (*4*,*5*,*7*,*8*) and included fever (93%), headache (43%), and arthralgia (40%), without hemorrhage. Renal dysfunction was found in ≈83% of patients, and approximately three-fourths of the patients were temporarily hospitalized (n = 29). Serologic investigations by ELISA and indirect immunofluorescence assay confirmed PUUV-reactive immunoglobulin M (IgM) and IgG antibodies in serum specimens from all 89 patients. The average age of the patients was 39 years (range 6–65 years), and the male/female ratio was 2.6:1.

For a large number of patients, the exposure to PUUV most likely occurred in a forested park and recreation area (“Stadtwald,” 20 ha) in Cologne’s inner city circle (Figure) where they lived, worked, or enjoyed recreational activities. Five patients had homes adjacent to this area. Four patients were evaluated for likely exposure due to employment at the RheinEnergie Stadium, 1 player in the German Football League and 3 employees who had cleaned basements or attics at the stadium (Figure). Three patients were members of a tennis club located near the stadium. To further investigate these cases, in April and June 2005, rodents were trapped in the Stadtwald. The effort yielded 35 bank voles, 17 yellow-necked mice, and 1 wood mouse. Screening of 48 available serum specimens by ELISA with yeast-expressed nucleocapsid (N) proteins of PUUV and DOBV (*9*) demonstrated 19 reactive blood samples. Seventeen had a higher endpoint titer to PUUV, and 2 showed identical endpoint titers for PUUV and DOBV. These 19 reactive samples (63%) originated from 30 *M. glareolus* bank voles.

**Figure:**
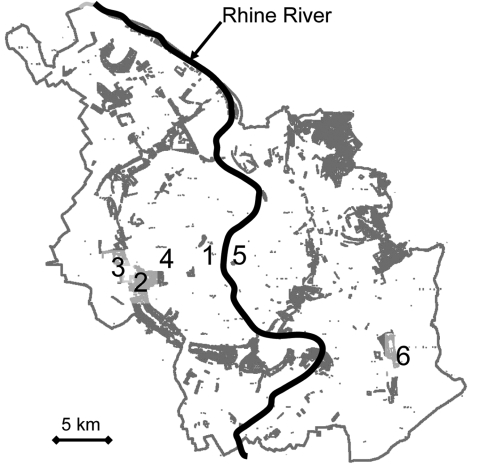
City of Cologne, showing its corridor of wooded public parks (shaded area) and the location of the exposure sites in the Stadtwald stadium area: 1, Cologne cathedral; 2, Stadtwald; 3, RheinEnergie Stadium; 4, university; 5, trade fair; 6, airport.

Lung tissues of all 53 mice were analyzed by PUUV-specific reverse transcription (RT)–PCRs targeting the S segment (for primers, see [*9*]). In 23 (66%) of the 35 bank voles, but in none of the other rodents, PUUV-specific RNA was amplified and sequenced. The concordance of ELISA– and RT-PCR–positive samples was 98% (Appendix Table).

Comparison of the partial S-segment nucleotide sequences obtained showed intersequence distances of 0%–1.2%. The level of the nucleotide sequence divergence from previously described German PUUV strains was 14.7%–16%. In phylogenetic analyses (neighbor-joining, maximum likelihood) based on this fragment, all sequences from the Cologne cluster formed a distinct group next to the branch consisting of strain Erft (95.4%–96% identical). These strains were clearly separated from additional PUUV strains originating from Germany (Berkel, southeastern Germany, Heidelberg), and Belgium, a neighboring European country (data not shown).

The only hantavirus known in an urban environment is Seoul virus, which is transmitted mainly by the peridomestic brown rat (*Rattus norvegicus*). As with most other hantaviruses, PUUV patients, including those previously observed in Germany, were reported to become infected in their rural residences or, when living in urban regions, during visits to the countryside in their spare time (*4*).

To our knowledge, this is the first report describing an outbreak of PUUV infections in a metropolitan area in Europe with a defined exposure site in the city center. The PUUV outbreak in 1990 in the city of Ulm occurred due to exposure during field military maneuvers in the outskirts of the city near the Danube River, and the surrounding civilian population did not experience a similar outbreak (*6*). The exposure site of a previous cluster of PUUV-infected patients reported from the city of Ulm and its surroundings remain uncertain but might be also rural because almost all patients lived outside the city of Ulm (*8*). In Cologne, about two thirds of the bank voles captured at the exposure site carried PUUV and are assumed to be the most probable source of infection. Increased sightings of rodents were reported by local health offices and pest control units. Studies at putative exposure sites in southeastern Germany in 2004 also showed a high prevalence of PUUV in the respective bank vole populations.

These cases are also the first indication, to our knowledge, that recreational activities in a forested city park, infested by hantavirus-infected rodents, may lead to human infections. This possibility should be investigated carefully in outbreak situations and may have practical implications for the future surveillance and prevention of NE in Europe.

## Supplementary Material

Appendix Table ELISA and RT-PCR results of the investigated rodent samples*
