# Supplementary material for: Nephropathia Epidemica in Metropolitan Area, Germany
Source: Emerg Infect Dis. 2007 Aug;13(8):1271–3. doi: 10.3201/eid1308.061425 (PMC2828076; doi:10.3201/eid1308.061425)
Supplement: Appendix Table — ELISA and RT-PCR results of the investigated rodent samples* [file 06-1425_appT-s1.pdf]

Appendix Table. ELISA and RT-PCR results of the investigated rodent samples*

	RT-PCR		
	Positive	Negative	NT
ELISA			
Positive	19	0	0
Negative	1	28	0
NT	3	2	0
	23	30	

*RT, reverse transcription; NT, not tested.
